# Beclin 1 of megakaryocytic lineage cells is locally dispensable for platelet hemostasis but functions distally in bone homeostasis

**DOI:** 10.1038/s41413-025-00410-7

**Published:** 2025-03-03

**Authors:** Lei Li, Chen Zhao, Ruizhi Zhang, Wen Wei, Bowen Liu, Jin Dong, Xueqin Gao, Di Zhang, Xueqing Wang, Meilin Lu, Yumu Zhang, Yao Yu, Na Yuan, Youjia Xu, Jianrong Wang, Yixuan Fang

**Affiliations:** 1 Research Center for Blood Engineering and Manufacturing, Cyrus Tang Medical Institute, Suzhou Medical College, Soochow University, Suzhou, China; 2 National Research Center for Hematological Diseases, State Key Laboratory of Radiation Medicine and Protection, Collaborative Innovation Center of Hematology, Institute of Blood and Marrow Transplantation, Jiangsu Institute of Hematology, The First Affiliated Hospital of Soochow University, Suzhou, China; 3 The Affiliated Ninth Suzhou Hospital of Soochow University, Suzhou, China; 4 Osteoporosis Institute, Department of Orthopedics, Second Affiliated Hospital of Soochow University, Suzhou, China

**Keywords:** Calcium and phosphate metabolic disorders, Bone

## Abstract

The crosstalk between megakaryocytic lineage cells and the skeletal system has just begun to be explored but remains largely elusive. Using conditional gene knockout mouse models, we demonstrated that loss of Beclin 1 (Becn1), a major regulator of mammalian autophagy, exclusively in the megakaryocytic lineage disrupted autophagy in platelets but did not compromise megakaryopoiesis or the formation and function of platelets. Unexpectedly, conditional *Becn1* deletion in male mice led to a remarkable increase in bone mass with improved bone quality, in association with a decrease in sex hormone binding globulin (SHBG) and an increase in free testosterone (FT). In vivo Becn1 overexpression in megakaryocytic lineage-specific cells reduced bone mass and quality, along with an increase in SHBG and a decrease in FT. Transplantation of wild-type bone marrow cells into megakaryocytic lineage *Becn1*-deficient male mice restored bone mass and normalized SHBG and FT. Furthermore, bilateral orchiectomy of *Becn1*^*f*/f^;*Pf4-iCre* mice, which are crippled with the production of testosterone, resulted in a reduction in bone mass and quality, whereas in vivo overexpression of SHBG, specifically in the liver of *Becn1*^*f*/f^;*Pf4-iCre* mice, decreased FT and reduced bone mass and quality. In addition, metformin treatment, which induces SHBG expression, reduced FT and normalized bone mass in *Becn1*^*f*/f^;*Pf4-iCre* mice. We thus concluded that Becn1 of the megakaryocytic lineage is dispensable locally for platelet hemostasis but limits bone mass by increasing SHBG, which in turn reduces the FT of male mice. Our findings highlight a mechanism by which Becn1 from megakaryocytic lineage cells distally balances bone growth.

## Introduction

Platelets originate from megakaryocytes (MKs) in the bone marrow (BM)^1^ and the lung.^2–4^ MKs undergo cytoplasmic maturation and polyploidization and release platelets into the circulation in response to blood flow.^5–9^ Canonically, MKs and platelets are associated primarily with maintaining hemostasis.^10,11^ However, increasing evidence has revealed that platelets are involved in various physiological and pathological processes, including the immune response.^11–14^ and cancer development.^15^ Early studies on platelet-rich plasma (PRP) have also indicated a potential role for platelets in bone health. Adding PRP to the graft increases the bone density of the recipient.^16^ PRP enhances the secretion of osteoprotegrin, an inhibitor of osteoclast formation, to inhibit osteoclast formation.^17^ The administration of PRP to animals significantly improved bone healing, tissue differentiation rates, and bone regeneration, as well as torsional stiffness, three-point load-bearing capacity, and overall bone strength.^18^ Furthermore, the sustained release of PRP along with bone morphogenetic protein 2-modified MSCs can significantly promote bone regeneration.^19^ PRP also plays a role in altering the matrix-forming phenotype of human mesenchymal stromal cells based on spatial configuration.^20^ Recent studies have reported the trans-regulation of bone homeostasis by megakaryocytic lineage cells, suggesting several mechanisms mediating such effects that involve MK-derived factors.^21–25^

Becn1 is a major member of the mammalian autophagy machinery and is implicated in various biological processes, including cellular metabolism, stress management, antitumorigenesis, antiviral host defense, and antiaging activities.^26–30^ In this study, we generated a conditional knockout mouse with biallelic *Becn1* deletion in megakaryocytic lineage cells, initially aiming to investigate the mechanistic impact of Becn1 on platelet function. Unanticipatedly, our findings revealed that while targeted deletion of Becn1 in megakaryocytic cells disrupts autophagy, it does not affect platelet hemostasis in either male or female mice. Instead, the deletion leads to increased bone mass and improved bone quality by promoting osteogenesis and inhibiting osteoclastogenesis, whereas in vivo overexpression of Becn1 exclusively in megakaryocytic cells resulted in the opposite phenotype, which is all mediated through alterations in SHBG and FT levels in the circulation of male mice. Our study suggested that Becn1, despite being considered a key player in autophagy, does not physiologically function locally in megakaryocytic lineage cells but does exert distant effects on balancing bone mass independent of autophagy.

## Results

### Biallelic *Becn1* deletion in the megakaryocytic lineage disrupted the expression of only four proteins relevant to autophagy in the proteome

**Fig. 1:**
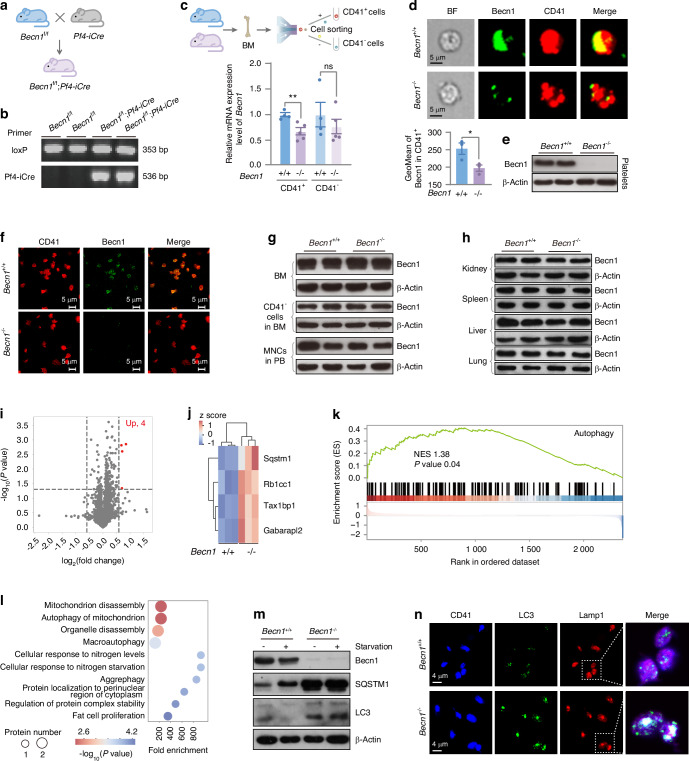
Generation of megakaryocytic lineage-specific *Becn1* knockout *Becn1*^*f*/f^;*Pf4-iCre* mice. **a**–**c** Generation of *Becn1*^*f*/f^;*Pf4-iCre* mice, identified by electrophoresis of tail DNA and analysis of *Becn1* mRNA expression levels by RT‒qPCR in CD41^**+**^ cells and CD41^–^ cells from the BM. *Gapdh* was used as a reference gene. **d** Analysis of Becn1 expression (green) in MKs from *Becn1*^f/f^ and *Becn1*^*f*/f^;*Pf4-iCre* mice by imaging flow cytometry. MKs were identified by CD41 staining. The upper panel shows a representative flow image, and the lower panel shows the results of the flow image analysis. **e** Analysis of the Becn1 protein expression level in platelets by western blotting. β-Actin was used as a loading control. **f** Analysis of Becn1 expression (green) in platelets from *Becn1*^f/f^ and *Becn1*^*f*/f^;*Pf4-iCre* mice by confocal microscopy. Platelets were labeled with CD41 (red). **g**, **h** Western blot analysis of Becn1 expression in whole BM cells, CD41^–^cells from the BM, and MNCs from the peripheral blood, kidney, spleen, liver, and lung. **i** Volcano plot of the platelet proteome. Differentially expressed proteins (DEPs) were determined under the conditions of |log_2_(fold change)| ≥ log_2_(1.5) and *P* < 0.05. **j** Heatmap of DEPs. **k** Gene set enrichment analysis (GSEA) of proteins involved in autophagy. The signaling pathway was adapted from the Gene Ontology (GO) pathway (GO:0006914). **l** GO enrichment analysis of DEPs in platelets. The ten most significant biological process (BP) terms. **m** Western blot analysis of autophagy-related proteins in platelets with or without starvation. **n** Colocalization analysis of LC3 and Lamp1 in platelets using confocal microscopy. Data are means ± SEMs. *P < 0.05; **P < 0.01

To explore the role of Becn1 in megakaryocytic lineage cells, *Becn1*^f/f^ mice^31^ were crossed with Pf4-iCre mice^32^ to obtain *Becn1*^*f*/f^;*Pf4-iCre* mice (Fig. 1a). Genotyping analysis confirmed the correct insertion of loxP sites and the presence of the Pf4-iCre sequence in the *Becn1*^*f*/f^;*Pf4-iCre* mouse (Fig. 1b). CD41^+^ (MKs and platelets) and CD41^−^ cells were separated from BM cells by fluorescence-activated cell sorting (FACS) (Fig. 1c, top), and the mRNA expression level of *Becn1* was quantified by quantitative reverse transcription polymerase chain reaction (RT‒qPCR). The results demonstrated a significant reduction in *Becn1* transcription in CD41^+^ cells, whereas CD41^−^ cells showed no change (Fig. 1c, bottom). We next employed imaging flow cytometry to examine Becn1 in bone marrow MKs. In line with the downregulation of the *Becn1* gene, the colocalization of Becn1 and CD41 was diminished in MKs from *Becn1*^*f*/f^;*Pf4-iCre* mice (Fig. 1d). The protein level of Becn1 in platelets was assessed using western blotting and confocal immunofluorescence assays. Becn1 was undetectable in platelets from *Becn1*^*f*/f^;*Pf4-iCre* mice (Fig. 1e, f). These findings indicate successful deletion of Becn1 in megakaryocytic lineage cells. To confirm the specificity of the knockout, Becn1 protein levels were examined in BM, CD41^−^ cells from the BM, mononuclear cells from peripheral blood (PB) and nonhematopoietic cells using western blotting. Importantly, no significant difference in Becn1 levels was detected between *Becn1*^f/f^ and *Becn1*^*f*/f^;*Pf4-iCre* mice in nonmegakaryocytic lineage cells (Fig. 1g, h). Collectively, these results demonstrated a marked reduction in Becn1 in MKs and nearly complete loss of Becn1 in platelets, confirming the successful generation of the conditional *Becn1* knockout mouse strain.

To investigate how the absence of Becn1 impacts the platelet proteome, proteomic profiling was conducted on platelets obtained from *Becn1*^f/f^ and *Becn1*^*f*/f^;*Pf4-iCre* mice. Differentially expressed protein analysis revealed that only four proteins were upregulated following *Becn1* deletion in platelets, as shown by the red dots (Fig. 1i). Interestingly, all four of these upregulated proteins are autophagy-related proteins (Fig. 1j), including Sqstm1/p62, an autophagy receptor;^33^ Rb1 cc1/FIP200/Atg17, which regulates autophagosome formation through direct interaction with Atg16L1;^34^ Tax1 bp1, which acts as the major recruiter of RB1CC1 to SQSTM1-ubiquitin condensates to promote their autophagic degradation;^35,36^ and Gabarapl2/Atg8/Atg8c, which are involved in the early and late stages of autophagy.^37,38^ Gene set enrichment analysis (GSEA) revealed significant changes in autophagy (GO:0006914) in platelets from *Becn1*^*f*/f^;*Pf4-iCre* mice (Fig. 1k). Furthermore, Gene Ontology (GO) enrichment analysis demonstrated that the functions of these differentially expressed proteins were mostly associated with autophagy (Fig. 1l).

To validate the proteomic data suggesting that Becn1 is involved in platelet autophagy, we examined autophagic flux in *Becn1*-deleted platelets. Western blot analysis revealed that the absence of Becn1 led to the inhibition of basal autophagy in platelets. This inhibitory effect was not reversed by platelet starvation, a potent trigger for autophagy activation, as confirmed by the results of western blotting and confocal microscopy (Fig. 1m, n). Hence, the absence of Becn1 in platelets disrupts autophagy without affecting protein expression in other biological processes in platelets.

### Biallelic *Becn1* deletion in the megakaryocytic lineage did not affect megakaryopoiesis

**Fig. 2:**
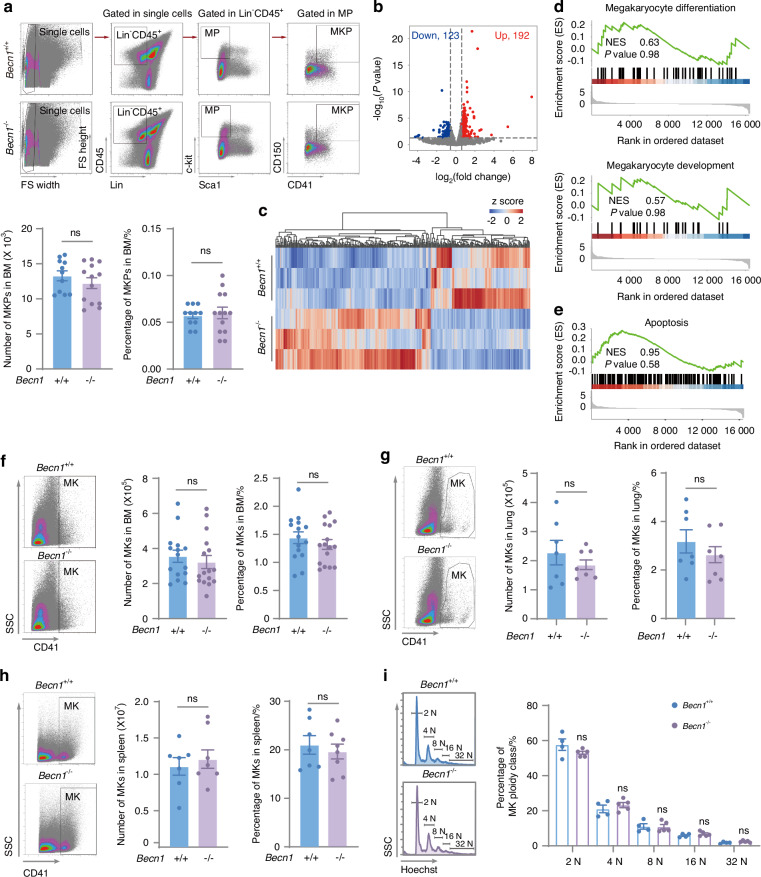
Pf4-iCre-mediated *Becn1* deletion does not impair megakaryopoiesis. **a** Number and percentage of MK progenitors (MKPs) in the bone marrow of *Becn1*^f/f^ and *Becn1*^*f*/f^;*Pf4-iCre* mice. Upper panel: Flow cytometric analysis of MKPs (Lin^–^CD45^+^Sca1^–^c-kit^+^CD150^+^CD41^+^). Lower panel: Statistical analysis of the number and percentage of MKPs in the bone marrow. MP cells: Lin^–^CD45^+^Sca1^–^c-kit^+^. **b** Volcano plot of MKs depicting the transcriptome of MKs. Differentially expressed genes (DEGs) were determined under the conditions of a |log_2_(fold change)| ≥ log_2_(1.5) and a *P* < 0.05. A total of 192 genes were upregulated, and 123 genes were downregulated. **c** Heatmap representing DEGs in the MK transcriptome. **d** GSEA of megakaryocyte differentiation (upper panel, GO:0030219) and megakaryocyte development (lower panel, GO:0035855). **e** GSEA of apoptosis (KEGG pathway, mmu04210). **f** Number and percentage of MKs in the bone marrow. Left panel: Flow cytometric analysis of MKs (CD41^+^) in the bone marrow. Right panel: Statistical analysis of the number and percentage of MKs in *Becn1*^f/f^ and *Becn1*^*f*/f^;*Pf4-iCre* mice. **g** Number and percentage of MKs in the lung. Left panel: Flow cytometric analysis of MKs in the lung. Right panel: Statistical analysis of the number and percentage of MKs in the lung. **h** Number and percentage of MKs in the spleen. Left panel: Flow cytometric analysis of MKs in the spleen. Right panel: Statistical analysis of the number and percentage of MKs in *Becn1*^f/f^ and *Becn1*^*f*/f^;*Pf4-iCre* mouse spleens. **i** Ploidy distribution of MKs in *Becn1*^f/f^ and *Becn1*^*f*/f^;*Pf4-iCre* mice. MK ploidy was measured by a double-staining technique (CD41 and Hoechst) and flow cytometry. Left panel: Histograms of DNA content (Hoechst) in MKs (CD41^+^). Right panel: Statistical analysis of MK ploidy levels. Data are means ± SEMs

MKs differentiate from megakaryocyte progenitors (MKPs). To determine whether Becn1 influences MK differentiation and maturation, we examined the number and percentage of MKPs (Lin^−^CD45^+^Sca1^−^c-kit^+^CD150^+^CD41^+^). Flow cytometric analysis revealed no significant change in the number or percentage of MKPs with *Becn1* deletion (Fig. 2a). Transcriptome sequencing of bone marrow MKs allowed us to evaluate the impact of *Becn1* deletion on the transcriptomic landscape. *Becn1*-deleted MKs were enriched in only 315 differentially expressed genes (DEGs), with 192 genes being upregulated and 123 genes being downregulated (Fig. 2b, c). GSEA revealed no alterations in megakaryocyte differentiation (GO:0030219) or development (GO:0035855) (Fig. 2d). The deletion of *Becn1* had no effect on the apoptosis of MKs (Fig. 2e). Furthermore, we observed no changes in the number or proportion of MKs in the BM (Fig. 2f), indicating that *Becn1* deletion did not affect MK production in this context.

In addition to the bone marrow, which is a major site of platelet generation, the lung also serves as a reservoir for platelet production,^4^ and the spleen is a principal organ for platelet destruction and removal.^39,40^ Consequently, we examined the number and percentage of MKs in the lung, which did not significantly change (Fig. 2g). The spleen is an important organ for extramedullary hematopoiesis.^41^ Like those in the BM and lung, the number and percentage of MKs in the spleen did not differ (Fig. 2h). Flow cytometric analysis of the DNA level indicated that the ploidy of *Becn1*-deleted MKs was not altered (Fig. 2i). Thus, the deletion of biallelic *Becn1* in the megakaryocytic lineage did not impair megakaryopoiesis in the BM, lung, or spleen.

### Megakaryocytic lineage-restricted deletion of *Becn1* did not affect platelet lifespan or function

To examine whether platelets become more sensitive to apoptotic triggers in the absence of Becn1, platelets from *Becn1*^*f*/f^;*Pf4-iCre* mice were exposed to ABT-737, a BH3 mimetic compound that enhances apoptosis sensitivity by counteracting antiapoptotic BCL-2 family proteins.^42^ Flow cytometric results with Annexin V staining demonstrated that the level of apoptosis in *Becn1* knockout platelets remained unchanged under both basal and apoptotic induction conditions compared with that in their wild-type counterparts (Fig. S1a). Spectrophotometric examination of caspase 3 activity, with or without the presence of an apoptotic activator, revealed no significant effect in the absence of Becn1 (Fig. S1b). Furthermore, western blot analysis revealed no significant differences in the dynamics of the expression of members of the apoptotic pathway, such as Bax, Bcl-xL, and Caspase 3, between Becn1-treated and Becn1-absent platelets in response to the basal or apoptotic inducer ABT-737 (Fig. S1c). Consistent with the biochemical results, a heatmap analysis of the proteomic data indicated that *Becn1* deletion did not affect apoptosis levels or the apoptotic pathway in platelets. No significant changes in the expression of apoptosis-related proteins were detected via platelet proteomics analysis (Fig. S1d).

**Fig. 3:**
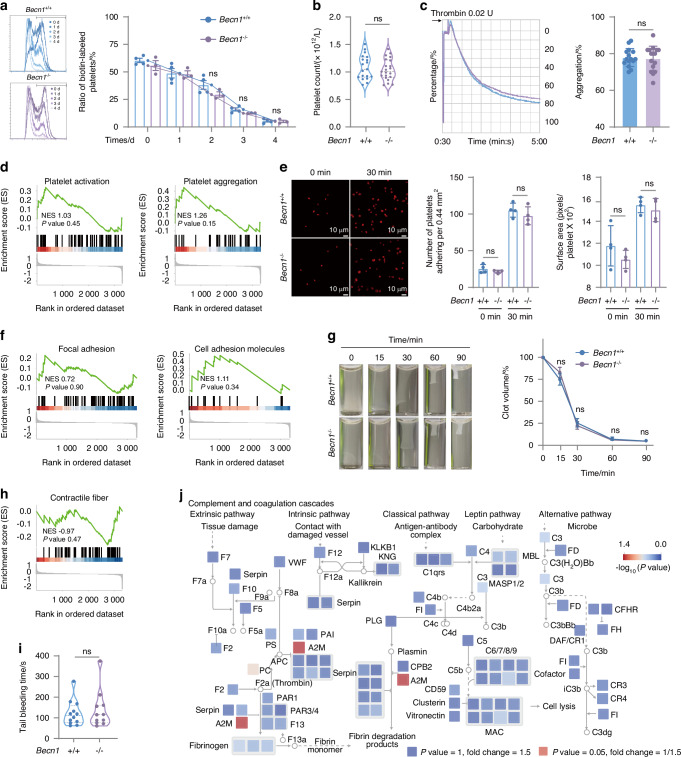
Pf4-iCre-mediated *Becn1* deletion does not impair the lifespan or function of platelets. **a** Flow cytometric analysis of the percentage of biotin-labeled platelets in *Becn1*^f/f^ and *Becn1*^*f*/f^;*Pf4-iCre* mice after the injection of NHS-biotin. **b** Platelet counts in *Becn1* ^f/f^ and *Becn1*^*f*/f^;*Pf4-iCre* mice. **c** Platelet aggregation levels in response to 0.02 U thrombin. Left panel: representative aggregation tracing of platelets using platelet aggregometry; right panel: statistical analysis of aggregation. **d** GSEA of platelet activation (GO:0030168) and aggregation (GO:0070527) terms. **e** Platelets spread on immobilized fibrinogen after treatment with 0.02 U of thrombin. Adherent cells were stained with phalloidin. Adhesion (number of platelets per 0.44 mm^2^) and spreading (mean platelet area) were visualized and evaluated via confocal microscopy and imaging. **f** GSEA of adhesion. Left panel: Focal adhesion (mmu04510). Right panel: Cell adhesion molecules (mmu04514). **g** Clot retraction of mouse platelets at different time points after treatment with 0.02 U thrombin. **h** GSEA of contractile fibers (GO:0043292). **i** Tail bleeding time of *Becn1*^f/f^ and *Becn1*^*f*/f^;*Pf4-iCre* mice. **j** The proteome changes observed in *Becn1*^f/f^ and *Becn1*^*f*/f^;*Pf4-iCre* mice were mapped to the KEGG pathway mmu04610, which formed a signaling network. The size of each node in the network was scaled to represent the magnitude of protein expression change in *Becn1*^f/f^ and *Becn1*^*f*/f^;*Pf4-iCre* mice. Furthermore, the color bar indicates the -log_10_(*P* value), which reflects the statistical significance of the differences between the two groups. Data are means ± SEMs

To investigate the role of Becn1 in platelet lifespan, biotin was injected into the tail vein of mice to label platelets. Blood samples were subsequently collected from the eye margin vein at various time points to measure the ratio of labeled platelets. The results indicated that the lifespan of platelets lacking Becn1 remained unchanged (Fig. 3a). Moreover, peripheral platelet counts did not differ between *Becn1*^f/f^ and *Becn1*^*f*/f^;*Pf4-iCre* mice (Fig. 3b). Platelet aggregation capacity, considered a “golden” indicator reflecting platelet function,^43^ was not inhibited in the knockout mice compared with the wild-type mice when stimulated with thrombin (Fig. 3c). GSEA of the platelet aggregation (GO database, GO:0070527) and platelet activation signaling pathways (GO database, GO:0030168) revealed no changes in either pathway (Fig. 3d). The results of the platelet adhesion assay, in which thrombin was added to the solution containing fibrinogen and incubated at 37 °C for 15 min, revealed no significant difference in the cytoskeleton or adhesion ability of platelets between the two groups of mice (Fig. 3e, f). Similarly, clot retraction of platelets was not affected in *Becn1* knockout mice compared with wild-type mice (Fig. 3g, h). Tail bleeding time was examined in *Becn1* knockout mice, and platelet hemostasis was not affected (Fig. 3i).

Consistent with the findings of the aforementioned laboratory tests, analysis of the proteomic data indicated that none of the proteins involved in the complement and coagulation cascades pathway from the KEGG database changed in the absence of Becn1 in platelets (Fig. 3j). These results indicate that the lifespan and function of platelets are not affected by the conditional deletion of *Becn1*.

### Nonmegakaryocytic lineage-specific deletion of *Becn1* impaired platelet formation and function

**Fig. 4:**
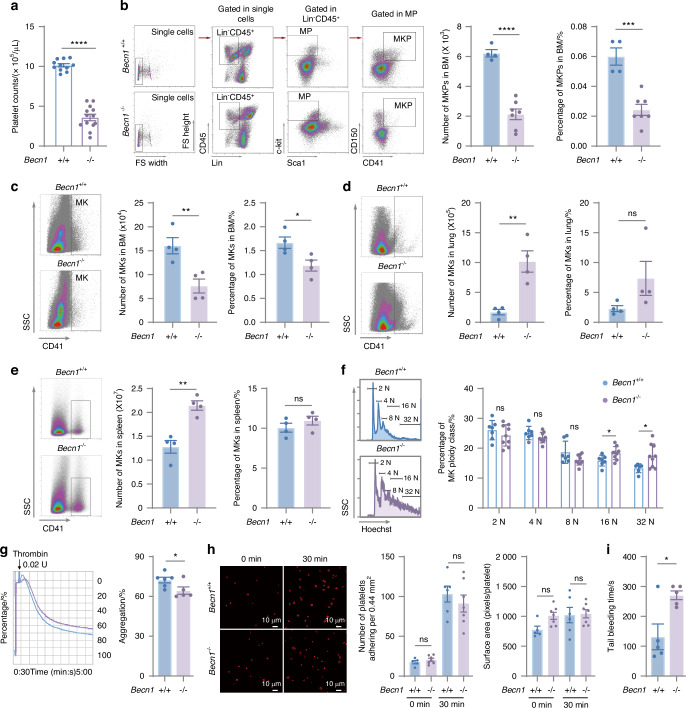
Mx1-Cre-mediated *Becn1* deletion impairs megakaryopoiesis and platelet function. **a** Platelet counts in the peripheral blood of *Becn1*^f/f^ and *Becn1*^*f*/*f*^;*Mx1-Cre* mice. **b** Number and percentage of MKPs in the bone marrow of *Becn1*^f/f^ and *Becn1*^*f*/*f*^;*Mx1-Cre* mice. Flow cytometric analysis of MKPs (left) and number and percentage of MKPs in the bone marrow (right). **c** Number and percentage of MKs in the BM. Flow cytometric analysis of MKs in the BM (left) and number and percentage of MKs in *Becn1*^f/f^ and *Becn1*^*f*/*f*^;*Mx1-Cre* mice (right). **d** Flow cytometric analysis of MKs in the lung (Left) and number and percentage of MKs in the lung (right). **e** Flow cytometric analysis of MKs in the spleen (left) and number and percentage of MKs in the spleens of *Becn1*^f/f^ and *Becn1*^*f*/*f*^;*Mx1-Cre* mice (right). **f** Ploidy distribution of MKs in *Becn1*^f/f^ and *Becn1*^*f*/*f*^;*Mx1-Cre* mice. Histograms of DNA content (Hoechst) in MKs (left) and percentage of MK ploidy class (right). **g** Platelet aggregation levels in response to 0.02 U thrombin. Representative aggregation tracing of mouse platelets using platelet aggregometry (left) and statistical analysis of the aggregation data (right). **h** Spread of 0.02 U of thrombin-treated platelets on immobilized fibrinogen. Adherent cells were stained with phalloidin. The adhesion (number of platelets per 0.44 mm^2^) and spreading (mean platelet area) of the platelets were visualized by confocal microscopy and evaluated by ImageJ. **i** Tail bleeding time of *Becn1*^f/f^ and *Becn1*^*f*/*f*^;*Mx1-Cre* mice. Data are means ± SEMs. *P < 0.05; **P < 0.01; ***P < 0.001; ****P < 0.000 1

Becn1 has been reported to be essential for platelet formation and function in whole-body constitutive monoallelic knockout mice (*Becn1*^+/-^), which exhibit prolonged bleeding time and weakened platelet aggregation; this biological significance is autophagy dependent.^44^ In contrast, our present study, which used a conditional gene knockout mouse model (*Becn1*^*f*/f^;*Pf4-iCre*), revealed that *Becn1* deletion exclusively in the megakaryocytic lineage does not compromise platelet formation or function in either male or female mice, although platelet autophagy is disrupted (Figs. 1–3). To determine whether nonspecific deletion of *Becn1* is the underlying cause of this discrepancy, a second mouse model with *Becn1* deletion in multiple tissues, including the entire hematopoietic lineage (*Becn1*^*f*/*f*^;*Mx1-Cre*), was generated, and a reduction in peripheral platelet counts was detected (Fig. 4a). *Becn1*^*f*/*f*^;*Mx1-Cre* mice also presented a reduction in the number and percentage of MK progenitors and MKs in the BM (Fig. 4b, c) but an increase in the number of MKs in the lung (Fig. 4d) and spleen (Fig. 4e). Flow cytometric analysis of DNA levels revealed a significant increase in the proportion of hyperploid MKs in the BM after Becn1 deficiency in the hematopoietic system (Fig. 4f). Overall, deletion of biallelic *Becn1* in the hematologic system impairs megakaryopoiesis in the BM and promotes extramedullary hematopoiesis in the lungs and spleen. *Becn1*^*f*/*f*^;*Mx1-Cre* mice displayed defective platelet aggregation in response to thrombin stimuli, as measured by lumi-aggregometry (Fig. 4g). Although the cytoskeleton and adhesion ability of the platelets were not altered (Fig. 4h), the duration of tail bleeding was prolonged (Fig. 4i). Our proteomic analysis revealed changes in only four proteins in the platelets of *Becn1*^*f*/f^;*Pf4-iCre* mice compared with those in the platelets of *Becn1*^f/f^ mice (Fig. 1). Both the number and function of platelets in *Becn1*^*f*/f^;*Pf4-iCre* mice remained normal (Fig. 3). Collectively, these results suggest that nonspecific deletions in cells of the hematopoietic system other than megakaryocytic lineage cells may be responsible for the impairment of platelet formation and function.

### Becn1 of the megakaryocytic lineage cells negatively regulates bone mass in males

**Fig. 5:**
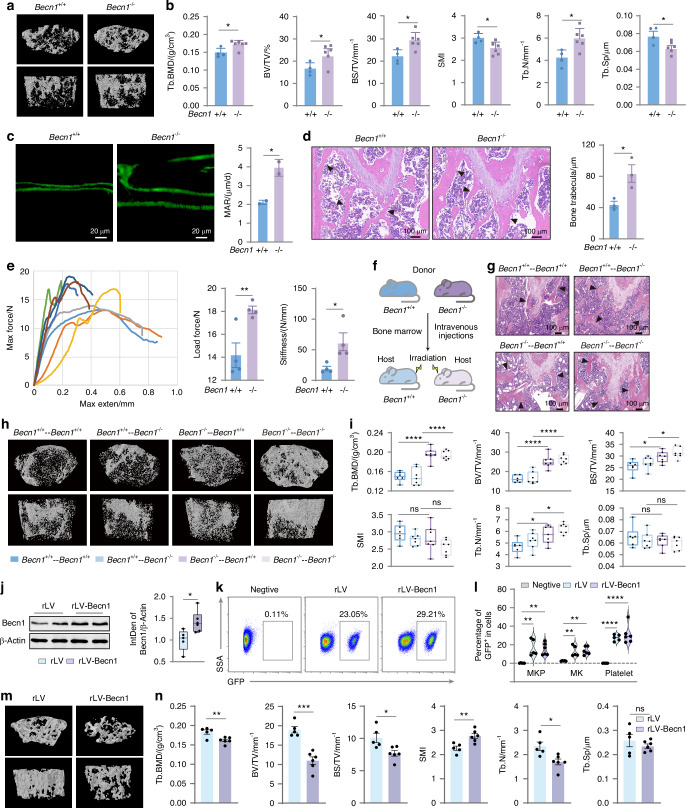
Becn1 of the megakaryocytic lineage cells negatively regulates bone mass in males. **a**, **b** Micro-CT analysis of *Becn1*^f/f^ and *Becn1*^*f*/f^;*Pf4-iCre* mice. **c** Representative calcein double-labeling images with quantification of the mineral apposition rate (MAR). **d** Representative image and statistical analysis of HE staining of the femur. **e** Three-point mechanical testing of bone strength and stiffness. **f** Diagram of the whole bone marrow transplantation mouse model. **g** HE staining of the femurs of transplant recipient mice (*Becn1*^+/+^−*Becn1*^+/+^, *n* = 2; *Becn1*^+/+^−*Becn1*^–/–^, *n* = 4; *Becn1*^–/–^−*Becn1*^+/+^, *n* = 2; *Becn1*^–/–^ −*Becn1*^–/–^, *n* = 3). **h**, **i** Micro-CT analysis of the transplanted mice. **j** Analysis of the Becn1 protein expression level in platelets by western blotting. β-Actin was used as a loading control. Left, representative western blot; right, statistical graph showing the relative expression levels of Becn1. **k** Percentages of GFP-positive platelets in rLV and rLV-Becn1 mice. **l** Statistical analysis of the percentages of GFP-positive MKPs, MKs, and platelets in rLV and rLV-Becn1 mice. **m**, **n** Micro-CT analysis of rLV and rLV-Becn1 mice. (rLV: Mice with megakaryocytic lineage-specific overexpression of the vector; rLV-Becn1: Mice with megakaryocytic lineage-specific overexpression of Becn1. Data are means ± SEMs. *P < 0.05; **P < 0.01; ***P < 0.001; ****P < 0.000 1

Three-dimensional (3D) reconstructions from micro-CT scans revealed greater bone mass and improved mineralization in male mice with Becn1 deletion in the megakaryocytic lineage (Fig. 5a). Statistical analysis of the micro-CT data of the distal femur trabecular bone structure indicated that male mice lacking Becn1, specifically in MKs and platelets, exhibited significantly greater bone mineral density (BMD), increased trabecular number (Tb.N), increased ratio of bone surface to tissue volume (BS/TV), and bone volume fraction (BV/TV), as well as a substantial reduction in both the structural model index (SMI) and the trabecular space (Tb.Sp), at 3 months of age (Fig. 5b). However, micro-CT scanning of the femur trabecular bones of female *Becn1*^*f*/f^;*Pf4-iCre* mice did not reveal any significant increase in bone mass (Fig. S2). Similarly, there were no notable changes in the BMD, BV/TV, BS/TV, Tb.N, SMI or Tb.Sp of the female mice (Fig. S2a). 3D reconstructions also revealed no significant changes in bone mass or mineralization in female mice lacking Becn1 in MKs or platelets (Fig. S2b). Consistent with these observations, calcein double labeling, which is used for measuring bone mineralization,^45,46^ revealed an increased rate of trabecular bone formation, especially in male mice lacking Becn1 (Fig. 5c). HE staining of the femur confirmed improved bone microstructure in male mice with *Becn1* deletion, as indicated by a notable increase in cancellous bone thickness (Fig. 5d). Additionally, a three-point bending test of bone biomechanical properties demonstrated enhanced bone quality in male mice lacking Becn1 (Fig. 5e).

To determine whether Becn1-absent platelets from male *Becn1*^*f*/f^;*Pf4-iCre* mice are responsible for the increase in bone mass, we performed a BM transplantation assay (Fig. 5f). HE staining of the femur confirmed an improvement in the bone microstructure of host mice that were transplanted with *Becn1*^*f*/f^;*Pf4-iCre* BM cells (Fig. 5g). 3D reconstruction of the micro-CT images revealed a substantial increase in bone mass in the recipient mice following the transplantation of the MK-deficient Becn1 mouse BM cells (Fig. 5h). Furthermore, the statistical analysis of the micro-CT results revealed greater BMD, increased Tb.N, greater BV/TV and BS/TV but no changes in either the SMI or the Tb.Sp after the transplantation of MK Becn1-deleted BM cells. (Fig. 5i).

To ensure that the increase in bone mass in male mice was not a result of nonspecific knockout of *Becn1* in bone tissue, we analyzed the protein levels of Becn1 in bone (Fig. S3). We used imaging flow cytometry to verify whether Becn1 was deleted nonspecifically in the upstream cells or progenitors of osteoblasts and osteoclasts, which are BM mesenchymal stem cells (MSCs) and macrophages, respectively. Image flow cytometric analysis revealed no reduction in Becn1 expression in the MSCs (Fig. S3a) or macrophages (Fig. S3b) of *Becn1*^*f*/f^;*Pf4-iCre* mice, excluding the nonspecific deletion of *Becn1* in the MSCs and macrophages. Western blotting revealed no significant changes in Becn1 expression in the bones of *Becn1*^*f*/f^;*Pf4-iCre* mice (Fig. S3c). Collectively, the above results demonstrate that the deletion of *Becn1* exclusively in MKs and platelets leads to increased bone formation and increased bone mass in male mice.

To determine the direct role of Becn1 of megakaryocytic lineage cells in regulating bone mass, we developed an in vivo model of MK-specific Becn1 overexpression via intramedullary injection of an rLV-GP1ba-Becn1-P2A-EGFP-WPRE virus, which resulted in increased Becn1 expression in platelets (Fig. 5j). Flow cytometry revealed a significant proportion of GFP-positive cells among MKPs, MKs, and platelets, further validating the megakaryocytic lineage-specific Becn1 overexpression model (Fig. 5k, l). 3D reconstruction of micro-CT images revealed a marked reduction in bone mass in mice with megakaryocytic lineage-specific Becn1 overexpression (Fig. 5m). Statistical analysis of the micro-CT data indicated a significant decrease in BMD, BV/TV, BS/TV and Tb.N, along with a notable increase in the SMI, in these mice (Fig. 5n).

Given that platelet autophagy was inhibited following the loss of Becn1 in platelets (Fig. 1m, n), we sought to determine whether the increase in bone mass in *Becn1*^*f*/f^;*Pf4-iCre* mice resulted from autophagic abnormalities by generating mouse models with platelet-specific deficiencies in Atg5 or Atg7, both of which are key autophagy proteins, via western blot analysis (Fig. S4a, d). 3D reconstruction of micro-CT images and statistical analysis of the micro-CT data revealed that the loss of Atg5 or Atg7 in platelets did not affect bone mass (Fig. S4b, c, e, f). These results suggest that Becn1 in megakaryocytic lineage cells regulates bone mass independently of autophagy.

### Becn1 of megakaryocytic lineage cells negatively regulates free testosterone levels, thereby inhibiting osteogenic activity but increasing osteoclastogenesis in males

**Fig. 6:**
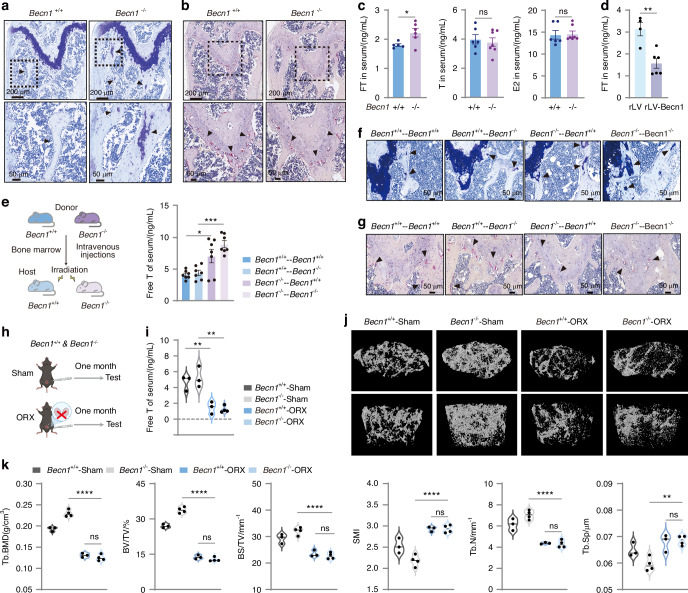
Becn1 of megakaryocytic lineage cells negatively regulates free testosterone levels, thereby inhibiting osteogenic activity but increasing osteoclastogenesis in males. **a** TB staining of mouse femurs (*Becn1*^f/f^, *n* = 3; *Becn1*^*f*/f^;*Pf4-iCre*, *n* = 3). **b** TRAP staining of mouse femurs (*Becn1*^f/f^, *n* = 3; *Becn1*^*f*/f^;*Pf4-iCre*, *n* = 3). **c** ELISA analysis of the serum levels of sex hormones. **d** ELISA analysis of free testosterone levels in the serum of rLV and rLV-Becn1 mice. **e** ELISA analysis of free testosterone levels in the serum of transplant recipient mice. **f** TB staining of the femurs of transplant recipient mice (*Becn1*^+/+^−Becn1^+/+^, *n* = 2; *Becn1*^+/+^−*Becn1*^−/−^, *n* = 4; *Becn1*^−/−^−*Becn1*^+/+^, *n* = 2; *Becn1*^−/−^−*Becn1*^−/−^, *n* = 3). **g** TRAP staining of the femurs of transplant recipient mice (*Becn1*^+/+^−*Becn*1^+/+^, *n* = 2; *Becn1*^+/+^−*Becn1*^−/−^, *n* = 4; *Becn1*^−/−^−*Becn1*^+/+^, *n* = 2; *Becn1*^−/−^−*Becn1*^−/−^, *n* = 3). **h** Schematic illustration of the bilateral orchiectomy (ORX) model. **i** ELISA analysis of serum testosterone levels in ORX mice. **j**, **k** Micro-CT analysis of ORX mice. Data are means ± SEMs. *P < 0.05; **P < 0.01; ***P < 0.001; ****P < 0.000 1

To investigate the underlying mechanism by which loss of Becn1 within the megakaryocytic lineage results in an increase in male bone mass, we used toluidine blue staining to characterize osteoblast activity in response to Becn1 deletion. The area of toluidine blue staining was significantly larger in the cancellous bone region of the femur (Fig. 6a), indicating a substantial increase in osteoblastic activity in *Becn1*^*f*/f^;*Pf4-iCre* mice. In parallel, TRAP staining of the *Becn1*^f/f^ and *Becn1*^*f*/f^;*Pf4-iCre* mice revealed a significant decrease in osteoclastic activity (Fig. 6b). These data suggest that *Becn1* deletion in megakaryocytic lineage cells influences male bone homeostasis by promoting osteogenesis and inhibiting osteoclastogenesis.

The absence of Becn1 in the megakaryocytic lineage led to enhanced bone formation in male but not female mice in our study (Figs. 5, S2), reminiscent of previous studies reporting that sex hormones regulate bone formation.^47,48^ This finding prompted us to hypothesize that the loss of Becn1 in the megakaryocytic lineage may influence sex hormones, ultimately resulting in increased bone formation. To test this hypothesis, we measured the levels of sex hormones in the serum of male *Becn1*^*f*/f^;*Pf4-iCre* mice. The results revealed a significant increase in free testosterone (FT) levels but no change in total testosterone or estradiol levels in male mice (Fig. 6c). In contrast, no alterations were detected in the serum levels of FT in *Becn1*^*f*/f^;*Pf4-iCre* female mice (Fig. S5a). This observation provides an explanation for the specific occurrence of increased bone mass solely in male mice following the deletion of *Becn1* in megakaryocytic lineage cells.

In the Becn1 overexpression model, we observed a significant decrease in serum FT levels following megakaryocytic lineage-specific overexpression of Becn1 (Fig. 6d). In the BM transplantation model, ELISA results confirmed a significant increase in FT levels in the serum of host mice transplanted with *Becn1*^*f*/f^;*Pf4-iCre* (Fig. 6e). Moreover, there was a significant increase in the extent of toluidine blue staining, which indicates greater osteoblastic activity, and a noticeable decrease in TRAP staining, which indicates less esteoclastic activity, in the femurs of host mice that received *Becn1*^*f*/f^;*Pf4-iCre* BM cells (Fig. 6f, g). Concurrently, bone formation was enhanced after the transplantation of MK lineage Becn1-deleted BM cells (Fig. 5h, i). These results suggest that the loss of Becn1 in MKs and platelets fosters oesteogenesis and inhibits osteoclastogenesis, presumably by increasing FT levels.

To determine the role of FT in the increase in bone mass in mice with megakaryocytic lineage-specific Becn1 deficiency, we established a bilateral orchiectomy (ORX) mouse model in *Becn1*^f/f^ and *Becn1*^*f*/f^;*Pf4-iCre* mice (Fig. 6h). The ELISA results revealed a significant decrease in the serum FT level in the mice after orchiectomy (Fig. 6i). 3D reconstructions revealed a significant reduction in bone mass in *Becn1*^*f*/f^;*Pf4-iCre* mice following orchiectomy (Fig. 6j). Micro-CT analysis revealed a significant decrease in the BMD, BV/TV, BS/TV and Tb.N, along with a significant increase in the SMI and Tb.Sp, in *Becn1*^*f*/f^;*Pf4-iCre* mice after orchiectomy (Fig. 6k). No significant difference in bone mass was observed between *Becn1*^f/f^ and *Becn1*^*f*/f^;*Pf4-iCre* mice after orchiectomy (Fig. 6k). These results suggest that Becn1 in megakaryocytic lineage cells regulates bone mass through the modulation of FT levels.

### Becn1 of megakaryocytic linease cells increases SHBG levels, leading to decreased release of free testosterone and reduced bone mass in males

**Fig. 7:**
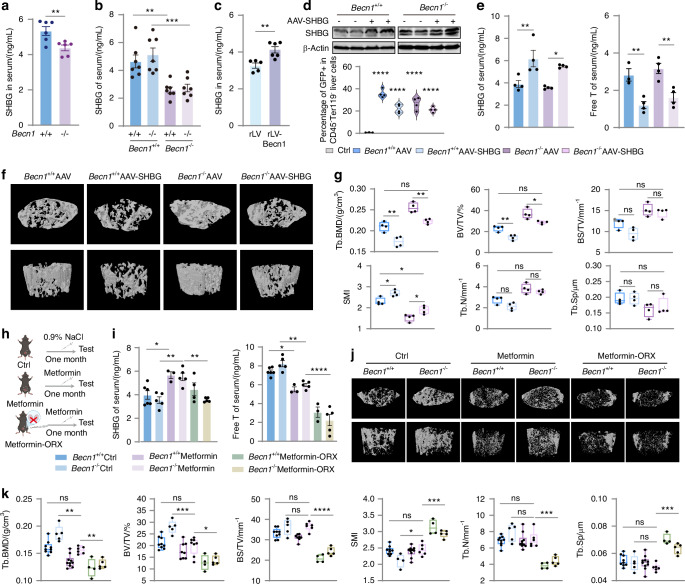
Becn1 of megakaryocytic linease cells increases SHBG levels, leading to decreased release of FT and reduced bone mass in males. **a** ELISA analysis of the serum levels of SHBG. **b** ELISA analysis of SHBG levels in the serum of transplant recipient male mice. **c** ELISA analysis of SHBG levels in the serum of rLV and rLV-Becn1 mice. **d** Western blot analysis of SHBG expression in liver tissue, with β-actin as a loading control. Upper panel, representative western blot; lower panel, statistical analysis of the percentage of GFP-positive cells in the CD45^–^Ter119^–^ liver population of the AAV and AAV-SHBG mice. **e** ELISA analysis of SHBG and FT levels in the serum of AAV and AAV-SHBG mice. **f**, **g** Micro-CT results for the AAV and AAV-SHBG mice. **h** Intervention diagram depicting metformin gavage treatment in Ctrl and ORX mice. **i** ELISA analysis of SHBG and FT levels in the serum of metformin-treated and ORX mice. **j**, **k** Micro-CT results of metformin-treated and ORX mice. Data are means ± SEMs. *P < 0.05; **P < 0.01; ***P < 0.001; ****P < 0.000 1

Sex hormone-binding globulin (SHBG) binds to circulating testosterone and regulates the levels of FT in the bloodstream.^49,50^ We hypothesized that Becn1 from megakaryocytic lineage cells may regulate FT via the regulation of SHBG. ELISA revealed a significant decrease in SHBG levels in male but not female *Becn1*^*f*/f^;*Pf4-iCre* mice (Figs. 7a, S5b). Furthermore, ELISA results confirmed a significant decrease in SHBG levels in the serum of host male mice transplanted with *Becn1*^*f*/f^;*Pf4-iCre* BM cells (Fig. 7b). On the other hand, in the Becn1 overexpression model, we observed a significant increase in serum SHBG levels following megakaryocytic lineage-specific overexpression of Becn1 (Fig. 7c). These data support the above hypothesis.

To determine whether SHBG reduction is responsible for the increase in bone mass in male *Becn1*^*f*/f^;*Pf4-iCre* mice, we established a mouse model with liver tissue-specific overexpression of SHBG. Western blot analysis confirmed a significant increase in SHBG protein levels in the liver tissue, and flow cytometry revealed a marked increase in the proportion of GFP-positive cells in the liver tissue (Fig. 7d), confirming the successful establishment of the liver tissue-specific SHBG overexpression model. The ELISA results revealed a significant increase in SHBG levels in the serum of SHBG-overexpressing mice, accompanied by a significant decrease in FT levels (Fig. 7e). 3D reconstruction revealed a significant reduction in bone mass in mice with liver tissue SHBG overexpression (Fig. 7f). Micro-CT analysis revealed a significant decrease in the BMD and BV/TV and a significant increase in the SMI, with no change in the Tb.N, BS/TV or Tb.Sp in *Becn1*^*f*/f^;*Pf4-iCre* mice after SHBG overexpression (Fig. 7g). No significant difference in bone mass was observed between *Becn1*^f/f^ and *Becn1*^*f*/f^;*Pf4-iCre* mice after SHBG overexpression, solely in *Becn1* knockout mice. These findings suggest that the loss of Becn1 in megakaryocytic lineage cells affects serum SHBG levels, thereby regulating bone mass.

To further determine whether Becn1 regulates bone mass via the SHBG-FT axis, we orally administered metformin, along with a combination of metformin treatment and orchiectomy, to increase the serum SHBG level in mice (Fig. 7h). The ELISA results demonstrated a significant increase in the serum SHBG levels following intervention with metformin in both *Becn1*^f/f^ and *Becn1*^*f*/f^;*Pf4-iCre* mice, accompanied by a significant decrease in FT levels (Fig. 7i). Concurrently, 3D reconstruction analysis revealed a substantial decrease in bone mass after metformin intervention or combined intervention with metformin and orchiectomy in both *Becn1*^f/f^ and *Becn1*^*f*/f^;*Pf4-iCre* mice (Fig. 7j). Moreover, micro-CT statistical analysis revealed a significant reduction in the BMD and BV/TV, a significant increase in the SMI, and no change in the Tb.N, BS/TV or Tb.Sp in *Becn1*^*f*/f^;*Pf4-iCre* mice following metformin intervention (Fig. 7k). In contrast, micro-CT statistical analysis revealed a significant decrease in the BMD, BV/TV, BS/TV and Tb.N, along with a significant increase in the SMI and Tb.Sp, in *Becn1*^*f*/f^;*Pf4-iCre* mice treated with a combination of metformin and orchiectomy (Fig. 7k). The results indicated that the SHBG increase was coupled with a decrease in FT and bone mass normalization in the *Becn1*-deleted group. These findings indicate that SHBG regulates bone mass by binding to FT.

Taken together, the increase in bone mass due to Becn1 deficiency in *Becn1*^*f*/f^;*Pf4-iCre* male mice is attributable to a decrease in SHBG and a subsequent increase in FT.

## Discussion

Autophagy has been implicated in bone metabolism.^51,52^ In particular, Becn1 plays a nonautophagic role in RANKL-induced osteoclastogenesis by inducing the production of reactive oxygen species and NFATc1.^53^ Loss of Becn1 in osteoclasts leads to a decrease in cancellous bone mass and an increase in cortical bone thickness in mice, accompanied by impaired chondrocyte differentiation.^54^ Mechanistically, TRAF6-mediated ubiquitination of Becn1 at K117 is a key step in RANKL-stimulated osteoclast differentiation. Mice lacking Becn1 in osteoclasts exhibited increased cortical bone thickness caused by impaired osteoclast function but also exhibited diminished trabecular bone mass, which was associated with defects in cartilage formation and chondrocyte differentiation.^54^ Becn1 was also implicated in the regulation of the apoptosis and differentiation of osteoclast precursors by IL17a, thus affecting osteoclast formation.^55,56^ Thus, Becn1 expressed in bone cells is indispensable for bone homeostasis because it regulates osteoclast and chondrocyte differentiation. Interestingly, a recent study reported that monoallelic deletion of Becn1 causes alterations in the local bone mechanical environment, including an increased response of bone adaptation to mechanical loading in female but not male Becn1^+/-^ mice, indicating that increasing cortical bone thickness significantly improves bone biomechanical behavior by effectively reducing stress and strain within the femoral shaft in female mice and suggesting that Becn1 functions on bone differently between sexes.^57^

Bone homeostasis can be regulated by factors outside of the skeletal system, such as vascular endothelial growth factor and endothelial cell-derived angiocrine factors,^58,59^ as well as liver-derived plasminogen.^60^ Mice with myelomonocytic cell-specific Ptpn11 deficiency exhibit mild osteopetrosis, suggesting a functional link between BM and bone health.^61^ Disruption of autophagy in the hematopoietic system accelerates bone aging and triggers osteoporosis, indicating reciprocal regulation between the hematopoietic system and the skeletal system via the autophagy pathway.^62^ MKs and platelets can also function beyond hemostasis, a canonical function of these cells.^63^ Specifically, the distal regulation of bone function by megakaryocytic lineage cells has been reported by two independent groups.^21,25^ MKs promote bone formation by linking osteogenesis, which enhances osteoblast proliferation and differentiation, with angiogenesis through the secretion of TGF-β1.^21^ Loss of IGF-I from MKs and platelets by Pf4-Cre reduces bone formation and regeneration without affecting BM adipogenesis, and PRP from *IGF1*^*f/f*^;*Pf4-Cre* mice has compromised osteogenic potential both in vitro and in vivo, suggesting that the therapeutic effects of PRP require IGF-1 from MKs and platelets and that megakaryocytic lineage cells are major sources of IGF-1 that distally coordinate to maintain and regenerate adult bone.^25^ Therefore, previous investigations have provided evidence of the positive impact of megakaryocytic lineage cells on bone formation through the secretion of cell factors such as TGF-β1 or IGF1.

Numerous studies have consistently highlighted the indispensable role of Becn1 in physiology.^64^ Using selective *Becn1*-deleted mice in the megakaryocytic lineage in *Becn1*^*f*/f^;*Pf4-iCre* mice, we demonstrated that Becn1 is dispensable for MKs and platelets. Nonmegakaryocytic lineage-specific deletion of Becn1 in *Becn1*^*f*/*f*^;*Mx1-Cre* mice impaired platelet formation and function (Fig. 4), which is consistent with the findings of a previous study in which whole-body constitutive monoallelic knockout mice (*Becn1*^+/^^−^) were used to conclude that universal deletion of Becn1 disrupts platelet formation and function.^44^ Therefore, these results suggest that nonspecific *Becn1* deletions in the hematopoietic system, but not specific *Becn1* deletions in megakaryocytic lineage cells, may be responsible for the impairment of platelet formation and function. These findings suggest that platelet formation and function may be regulated by Becn1 from nonmegakaryocytic lineage cells.

**Fig. 8:**
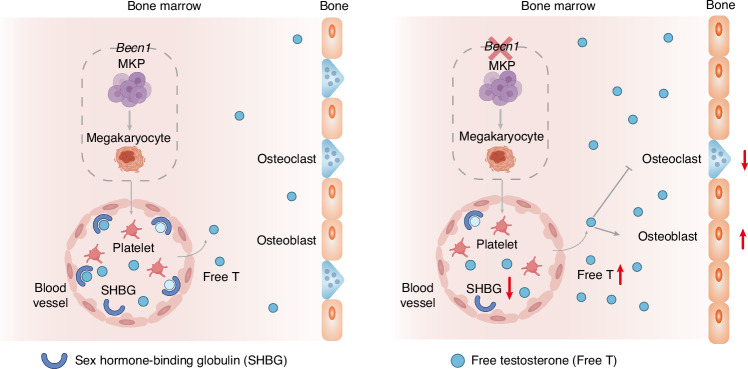
A cartoon illustrating the role of Becn1 of MK lineage cells in distally balancing bone mass. Becn1 from MK lineage cells from male mice increases the level of SHBG, thus reducing the amount of FT in the blood and ultimately limiting bone mass by inhibiting osteogenesis and enhancing osteoclastogenesis

More unexpectedly, our present work revealed that specific Becn1 deficiency in the megakaryocytic lineage increases bone mass and quality by promoting osteoblastic activity and inhibiting osteoclastic activity solely in male but not female mice. Intriguingly, loss of Becn1 in the megakaryocytic lineage reduces SHBG levels and increases FT levels in the circulation, whereas transplantation of wild-type BM cells increases SHBG levels, decreases FT levels in the blood and restores altered bone homeostasis in Becn1-absent male mice. In vivo Becn1 overexpression exclusively in megakaryocytic lineage-specific cells reduced bone mass and quality, accompanied by an increase in SHBG and a decrease in FT. Moreover, orchiectomy of *Becn1*^*f*/f^;*Pf4-iCre* mice, which crippled with the production of testosterone, led to a reduction in bone mass and quality, whereas in vivo overexpression of SHBG, specifically in the liver of *Becn1*^*f*/f^;*Pf4-iCre* mice, decreased FT and reduced bone mass and quality. Additionally, increasing SHBG levels via the administration of metformin reduces FT levels in the blood and restores bone homeostasis in Becn1-absent mice. Our results are consistent with previous reports that testosterone and estradiol are predominantly bound to other proteins in circulation, particularly SHBG.^49,50^ Thus, although Becn1 does not function locally in MKs and platelets, it distally regulates bone growth by influencing SHBG and FT levels in the blood (Fig. 8). Our findings highlight a previously undescribed mechanism by which megakaryocytic lineage cells regulate bone homeostasis and provide a new perspective for improving bone health, potentially by targeting Becn1 in the megakaryocytic lineage.

Several studies have demonstrated distal modulation of bone remodeling by megakaryocytic lineage cells. MKs contribute to bone formation by coupling osteogenesis with angiogenesis through the secretion of TGF-β1.^21^ IGF-1 from MKs, platelets and BM stromal cells promotes osteogenesis and inhibits adipogenesis.^25^ These studies establish that TGF-β1 and IGF-1, which are derived from MKs and platelets, play regulatory roles in bone homeostasis. In humans, an increase in pleomorphic MKs in the BM often leads to the development of osteosclerosis, whereas in mice, an increase in the number of MKs results in osteosclerosis in *GATA*-*1*^*low*^, *Nf*-*e2*^−/−^, *TPO*^*high*^, *Mpl*^*f*/*f*^;*PF4*-*cre*, *Lnk*^−/−^, *Mpig6b*^−/−^, *Mpig6b*^*f*/*f*^;*Gp1ba*^-^*Cr*^+^/*KI*, and PT-vWD genetically modified models.^65^ Recent studies have revealed that the ability of MKs to communicate with bone cells is affected by the age and sex of the animal.^65^ In particular, null and loss-of-function mutations in MPIG6B, an inhibitory heparan sulfate receptor G6b-B specific to MKs, cause severe macrothrombocytopenia, and female Mpig6b null mice present with elevated BM MK numbers and very high increases in bone mass, whereas males lose bone mass, and estrogen contributes to the severity of MK-driven osteosclerosis.^22^ Myeloproliferative neoplasms (MPNs) in mice and humans impact bone density and osteoblast proliferation and differentiation. The mechanisms responsible for MPN-associated changes in bone health involve MKs and their secreted factors.^23^ Lysyl oxidase (LOX) facilitates extracellular matrix cross-linking. In MK-specific *LOX* knockout mice, LOX expressed in MKs affects bone volume and collagen architecture in a sex-dependent manner.^24^ In our study, we found that the manipulation on Becn1 levels in megakaryocytic lineage cells caused an alteration in bone mass in male, but not female, mice via the modulation of SHBG and FT levels. Thus, proteins produced by MKs and platelets may mediate highly sex-specific osteogenic cells involving sex hormones. Further research is warranted to elucidate how Becn1 regulates SHBG-FT axis to distally influence bone homeostasis at the molecular level.

## Materials and methods

### Mice

MK and platelet-derived Becn1-deficient mice (*Becn1*^*f*/f^;*Pf4-iCre*) were generated by crossing Pf4-iCre mice (Jackson Laboratory) with Becn1-flox mice.^31^ Genotyping was performed by PCR analysis of tail DNA samples with specific primers amplifying the LoxP sites (F1-CGTTGGCTACCCGTGATATT, F2-TTTGTTTTGTGGGGAATTCATTGT, R-TGAGTAGTTATCTGGGCTGGGAGA). The presence of the iCre sequence was confirmed using the primers F-AAGCACATCACCAGCCTGGAG and R- GTTGTTCAGCTTGCACCAGG. The mice were bred and housed in specific-pathogen-free animal facilities at Soochow University. All animal experiments were approved by the institutional animal care and use committee of Soochow University.

### Reagents

The reagents used in this study are described in Table S1.

### Platelet preparation

The collected whole blood, with anticoagulant plus 1 μmol/L PGE-1 (Enzo Life Sciences, USA), was centrifuged at 800 r/min for 10 min at room temperature (RT) with no brake applied to prevent platelet activation. The supernatant was then gently transferred to a fresh tube using a polypropylene Pasteur pipette and centrifuged at 800 r/min for 10 min to obtain PRP. Washed PRP platelets were collected by centrifugation at 1 300 r/min for 15 min at RT and resuspended in modified Tyrode’s buffer (pH 7.4) containing 137 mmol/L NaCl, 20 mmol/L HEPES, 1 mmol/L MgCl_2_ ∙ 6H_2_O, 2.7 mmol/L KCl, 3.3 mmol/L Na_2_HPO_4_ ∙ 2H_2_O, 5.6 mmol/L D-glucose, and 1 g/L BSA.

### Flow cytometry

Washed platelets (3 × 10^8^/mL) from the mice were pretreated with ABT737 (5 μmol/L) or vehicle control for 2 h at 37 °C, and then, FITC-Annexin V and binding solution were added to the pretreated platelets at a ratio of 1:100 or 1:10 by volume. The platelet mixtures were mixed evenly and gently, incubated for 20 min at RT in the dark, and then subjected to flow cytometry analysis as described previously. BM cells were collected from femurs and tibiae. Flow cytometry-based cell sorting and analysis were performed using FACSAriaTM III (BD, USA) and Beckman Coulter Gallios (Beckman, USA).

### RNA extraction and RT‒qPCR

Total RNA was extracted from MKs using the MicroElute Total RNA Kit (Omega Biotek, USA) according to the manufacturer’s instructions. Reverse transcription (RT) was performed with total RNA using HiScript III RT SuperMix for RT-PCR (Vazyme, China). PCR was carried out using cDNA obtained from RT and 2x SYBR Green qPCR Master Mix (Vazyme, China) according to the manufacturer’s instructions.

### Western blotting

BM cells, platelets, and other organs were lysed in 1X cell lysis buffer (CST, USA) supplemented with protease and phosphatase inhibitors (Roche, Switzerland). Proteins were resolved by 12% SDS‒PAGE and transferred to PVDF membranes. The membranes were blocked with 5% skim milk-TBS-0.1% Tween 20 for 1 h at RT. Antibodies against Becn1, Bax (CST, USA), microtubule-associated protein 1A/1B-light chain 3 (Novus Biologicals, USA), Bcl-XL (CST, USA), P62 (MBL, USA), Caspase 3 (CST, USA), SHBG (HuaBio,China) and β-Actin (CST, USA) were used to probe the membranes. The membranes were then washed five times in TBST and incubated with HRP-conjugated secondary antibodies (anti-mouse or anti-rabbit, CST, USA) diluted 1:2 000 in TBST for 1 h. After five washes, the membranes were developed using an enhanced chemiluminescence kit (Biological Industries, Kibbutz Beit-Haemek, Israel).

### Immunofluorescence assay

Platelets were fixed in 4% paraformaldehyde for 15 min and permeabilized in 0.5% Triton X-100 for 5 min. The cells were subsequently incubated with an antibody overnight at 4 °C after being blocked with 4% bovine serum albumin for 60 min. The cells were treated with a secondary antibody and DAPI before being photographed under a fluorescence microscope (FV3000, Olympus, Japan).

### Caspase-3 assay

Caspase-3 activity was analyzed via a caspase-3 activity kit as described previously. Briefly, washed platelets (3 × 10^8^/mL) from mice were pretreated with ABT737 (5 μmol/L, Selleck, USA) or vehicle control for 2 h at 37 °C. The caspase-3 activity assay was performed on 96-well microtiter plates by adding 50 μL of platelet lysate per sample to 40 μL of assay buffer and 10 μL of caspase-3 substrate (Ac-DEVD-pNA, 2 mmol/L, DDJINDO, Japan). The samples were further incubated at 37 °C for 2 h, after which the absorbance at 405 nm was measured with an ELISA reader.

### Platelet life span analysis

The platelet life span assay and adoptive platelet transfer assay were conducted as described previously. Briefly, the mice were injected intravenously with N-hydroxysuccinimide ester (NHS)-biotin (300 mg per 20 g mouse, Sigma, Germany). Whole blood was collected from the orbital vein at various time points (0, 1, 2, 3, and 4 days), and platelets were isolated and prepared. The cells were stained with FITC-conjugated mouse anti-CD41 (BioLegend, USA) and PE-streptavidin (BioLegend, USA), and then flow cytometry was used to analyze the platelet life span.

### Bleeding time analysis

The bleeding time assay was performed using a razor blade to transect the mouse tail 3 mm from the tip, with the tail immersed in a 12 mL test tube containing PBS at 37 °C. Bleeding times were determined when the bleeding stopped for more than 10 s. If the bleeding time was longer than 15 min, the assay was stopped, and the bleeding time was 15 min. The control and experimental groups were matched for age, sex, and body weight.

### Platelet aggregation

The number of washed platelets was normalized to a final concentration of 2–4 × 10^8^ cells/mL with Tyrode’s buffer. Diluted washed platelets (250 µL) were placed in an aggregometer cuvette, warmed to 37 °C, and measured in a dual-channel platelet aggregation apparatus (Chrono-log, USA). After 1 mmol/L CaCl_2_ was added, baseline measurements were obtained, and 0.012 U/mL thrombin (Chrono-log, USA) was added to induce aggregation. The percent aggregation was calculated from the amplitude of the tracings at 5 min and normalized to the response of the untreated control within an individual experiment.

### Platelet adhesion assay

Platelets (2 × 10^7^ platelets/mL) prepared from the blood of the mice were incubated on Fg (100 µg/mL; Cayman, Hamburg, Germany)-coated coverslips (blocked with 1% BSA) for 60 min at 37 °C. Nonadherent platelets were removed, and the coverslips were washed with PBS before fixation with 4% paraformaldehyde. Platelets were permeabilized with 0.1% (v/v) Triton X-100 prior to staining with 0.1% (v/v) Alexa 594-conjugated phalloidin (Abcam, Cambridge, UK) for 60 min, and the coverslips were washed with PBS. Subsequently, adherent platelets were imaged with a 100× magnification oil immersion lens using a Leica SP5 II laser scanning confocal microscope equipped with a Leica DFC365 FX digital camera (Leica Microsystems, Heidelberg, Germany). Platelet surface areas were quantified via ImageJ software (NIH, USA).

### In vivo overexpression

For the in vivo MK lineage-specific *Becn1* overexpression model, the mice were anesthetized with tribromoethanol, and their knees were flexed with support behind each knee. The hair around the joint area was shaved, and the region was cleaned with 75% alcohol and iodine. A 1 mL syringe was inserted into the femur‒tibia joint through a puncture at the top of the femur between the condyles, with gentle twisting and pressure applied. After removal, 5 μL of viral solution (BrainVTA, China) was injected into the BM cavity using an insulin needle. Mice were monitored and allowed to recover, with assessments conducted 2 months post-injection. As a control, *Becn1*^f/f^ male mice were injected with the rLV-GP1ba-EGFP-WPRE virus into the BM cavity. The rLV-GP1ba-Becn1-P2A-EGFP-WPRE virus was injected to specifically overexpress Becn1 in megakaryocytic lineage cells.

For in vivo liver specific *SHBG* overexpression model, mice were secured in a tail vein injection apparatus with their tails exposed. The tail was cleaned with 75% alcohol, and 200 μL of diluted viral solution was injected via the tail vein using an insulin needle. Assessments were conducted 2 months postinjection. As a control, the rAAV-TBG-EGFP-WPRE-PA virus was injected into the tail vein of both *Becn1*^f/f^ and *Becn1*^*f*/f^;*Pf4-iCre* male mice. Conversely, the rAAV-TBG-Shbg-P2A-EGFP-WPRE-PA virus was injected to specifically overexpress SHBG in liver tissue.

### Orchiectomy model

At 9 weeks of age, both male *Becn1*^f/f^ and *Becn1*^*f*/f^;*Pf4-iCre* mice were subjected to ORX under tribromoethanol (Sigma, Germany) anesthesia followed by carprofen analgesia (50 µg/kg, Sigma, Germany). The sham group underwent anesthesia, suturing, and analgesia, without the removal of the testes. One month after surgery, samples were collected, and micro-CT scans were performed.

### Blood clot contraction test

Briefly, platelets (2 × 10^8^ platelets/mL) prepared from the blood were preincubated with various concentrations of CoQ_10_ or control solvent for 50 min and then added to glass tubes containing 200 µg/mL Fg and 1 mmol/L Ca^2+^. Clot retraction was initiated by adding 0.1 U/mL thrombin. Clot retraction was monitored every 15 min for 90 min at 37 °C and documented photographically using a Nikon camera.

### Micro-CT

The femurs were scanned and analyzed for trabecular bone by micro-CT (SkyScan 1174, Bruker, Kontich, Belgium). The acquisition parameters were as follows: X-ray voltage = 50 kV, X-ray current = 800 μA, filter = 0.5 mm aluminum, rotation step = 0.7°, and image pixel size = 10.3 μm. After scanning, images were reconstructed using NRecon software (Bruker, Kontich, Belgium). Parameters of trabecular bone were determined using CTAn software (Bruker, Kontich, Belgium), and 3D image reconstruction was performed using CTvOX software (Bruker, Kontich, Belgium). For trabecular bone parameters, the volume of interest (VOI = 100 slices) was selected with reference to the distal growth plate. The trabecular bone regions started ~0.7 mm and 5 mm from the growth plate and extended toward the proximal end of the femur. The cancellous bone parameters of the femoral metaphysis included the trabecular BMD, bone volume-to-total tissue volume ratio (BV/TV), bone surface-to-total tissue volume ratio (BS/TV), SMI, Tb.N, and trabecular spacing (Tb.Sp).

### Biomechanical properties

The biomechanical properties of the femur were measured via three-point bending and compression testing. Femur were collected and stored at −20 °C. Femur were tested via three-point bending with the posterior surface on the lower supports (5 mm apart), and the load was applied to the anterior surface centered between the lower supports, as previously described in detail (Akhter, Cullen, Gong, & Recker, 2001). Biomechanical structural strength variables, including the ultimate load and stiffness, were measured.

### Calcein labeling

Mice were intraperitoneally injected with 10 mg/kg calcein in a 1% saline solution for 10 or 3 days before they were sacrificed. The femurs were fixed overnight in 4% paraformaldehyde, dehydrated in 30% sucrose for 2 days, and sectioned for calcein labeling.

### Image flow analysis

Image flow cytometric analysis of the expression of Becn1 (NOVUS, USA) in MKs, macrophages and MSCs was performed with an image flow cytometer (Amnis, Merck Millipore). BM cells were fixed and permeabilized after staining for cell markers and labeled with antibodies against Becn1. The samples were visualized and analyzed for the expression of marker proteins with IDEAS 6.0 software (Amnis, Merck Millipore).

### Bone marrow transplantation assay

A total of 1 × 10^7^ cells were harvested from the BM of *Becn1*^f/f^ and *Becn1*^*f*/f^;*Pf4-iCre* male mice. The cells were injected into lethally irradiated (X-ray, 9 Gy) *Becn1*^f/f^ and *Becn1*^*f*/f^;*Pf4-iCre* male mice through the tail vein. The BMD of the recipient mice was monitored for up to 2 months after transplantation.

### Bone histology and immunohistochemistry

The fixed femoral bones were decalcified in 10% EDTA for 3 weeks, dehydrated in a graded ethanol series (70%–100%), cleared in xylene, and paraffin-embedded with the long axis of the bone parallel to the base plane to preserve anatomical orientation. Longitudinal serial 4 μm thick sections were cut and mounted on polylysine-coated microscope slides and subjected to hematoxylin and eosin, toluidine blue, and TRAP staining following the manufacturer’s instructions (Servicebio Science & Technology).

### RNA sequencing analysis

Shenzhen BGI Genomics (BGI, China) conducted RNA extraction and cDNA library construction on mouse MKs from 3-month-old mice, followed by sequencing using the DNBSEQ platform. Reads that were of low quality, contaminated with adapters, or had a high content of unknown base were filtered out. The HISAT program was used for reference genome mapping. Differential gene analysis was performed using DESeq2.^66^ Genes with fold change ≥ 1.5 and *P* < 0.05 were considered upregulated, whereas genes with fold change ≤ 1/1.5 and *P* < 0.05 were considered downregulated. The bioinfokit package was used to calculate the logarithm of the protein’s differential fold change, with a base of 2, as well as the absolute value of the logarithm of the *P* value, with a base of 10, subsequently resulting in the creation of a volcano plot.^67^ Furthermore, a cluster heatmap depicting the DEGs was generated. The GO and KEGG databases were utilized for GSEA of the identified genes, and gseaplot2 was used for pathway visualization.^68^

### Proteomic profiling

Beijing Novogene Corporation (Novogene, China) extracted mouse platelet proteins and performed TMT-based quantitative proteomics. Proteome Discoverer software was utilized for database retrieval, spectrum peptide analysis, and protein quantification. A further refinement process was subsequently executed through Proteome Discoverer software: peptide spectrum matches (PSMs) with a confidence level above 99% were considered reliable, and proteins containing at least one unique peptide were deemed reliable as well. Only trustworthy PSMs and proteins were retained, and false discovery rate (FDR) verification was implemented to eliminate peptides and proteins with an FDR greater than 1%. A comprehensive set of quality controls was then conducted, encompassing the distribution of peptide length, parent ion mass tolerance, unique peptide counts, protein coverage, and protein molecular weight. As an initial step, Proteome Discoverer utilized the original spectrum peak area to calculate the relative quantification values of each PSM in every sample. Next, the quantification information from all PSMs contained within the identified unique peptides was used to calibrate the relative quantification values of these peptides. The relative quantification values of each protein were subsequently calibrated by considering the quantification information of all unique peptides within that protein. The relative quantification values of proteins from the two groups were subjected to a *t* test to compute the corresponding *P* values. Proteins exhibiting fold change ≥ 1.5 and a *P* < 0.05 were considered upregulated, whereas proteins displaying fold change ≤1/1.5 and a *P* < 0.05 were considered downregulated. To visualize these findings, the bioinfokit package was used to calculate the logarithm of the protein fold change using a base of 2, as well as the absolute value of the logarithm of the *P* value using a base of 10, resulting in the creation of a volcano plot. Furthermore, a cluster heatmap was generated to visualize the differentially expressed proteins. For the analysis of measured proteins, GSEA via ClusterProfiler was performed based on the GO and Kyoto Encyclopedia of Genes and Genomes (KEGG) databases,^69^ whereas gseaplot2 was used for visualization of the autophagy pathway. Additionally, functional enrichment analysis of the differentially expressed proteins was conducted via the GO database, and a bubble plot was generated via matplotlib. Moreover, by utilizing pathview and matplotlib in conjunction with the KEGG database, the fold change and *P* value of all measured proteins within specific pathways were represented visually.

### Statistical analysis

Statistical analyses were performed using SPSS version 22.0. An unpaired *t* test was used to compare two sets of data between different groups, whereas one-way analysis of variance (ANOVA) was used when comparing three or more sets of data among different groups. Compare the mean of each column with the mean of a control column (Dunnett’s multiple comparisons test was used adjust *P* value). Compare the mean of each column with the mean of every other column (Tukey’s multiple comparisons test was used adjust *P* value). The data are expressed as the mean ± standard error of the mean (SEM). *P* < 0.05 was considered to indicate a statistically significant difference.

## Supplementary information

Supple data

## Data Availability

Proteome profiles are available via ProteomeXchange with identifier PXD058230. RNA-Seq data were deposited in the NCBI’s Gene Expression Omnibus data¬base under accession number GSE283093.
