# Supplementary material for: Beclin 1 of megakaryocytic lineage cells is locally dispensable for platelet hemostasis but functions distally in bone homeostasis
Source: Bone Res. 2025 Mar 3;13:32. doi: 10.1038/s41413-025-00410-7 (PMC11876339; doi:10.1038/s41413-025-00410-7)
Supplement: Supplementary file 1 — Supple data [file 41413_2025_410_MOESM1_ESM.pdf]

Supplementary material

Beclin 1 of megakaryocyte lineage cells is locally dispensable for platelet hemostasis but functions distally in bone homeostasis

Lei Li[1,2,4,#], Chen Zhao[1,2,#], Ruizhi Zhang[3,#], Wen Wei[1,2,4], Bowen Liu[1,2], Jin Dong[1,2], Xueqin Gao[1,2], Di Zhang[1,2], Xueqing Wang[1,2], Meilin Lu[1,2], Yumu Zhang[1,2], Yao Yu[1,2], Na Yuan[1,2,4], Youjia Xu[3,*], Jianrong Wang[1,2,4,*] and Yixuan Fang[1,2,4,*]

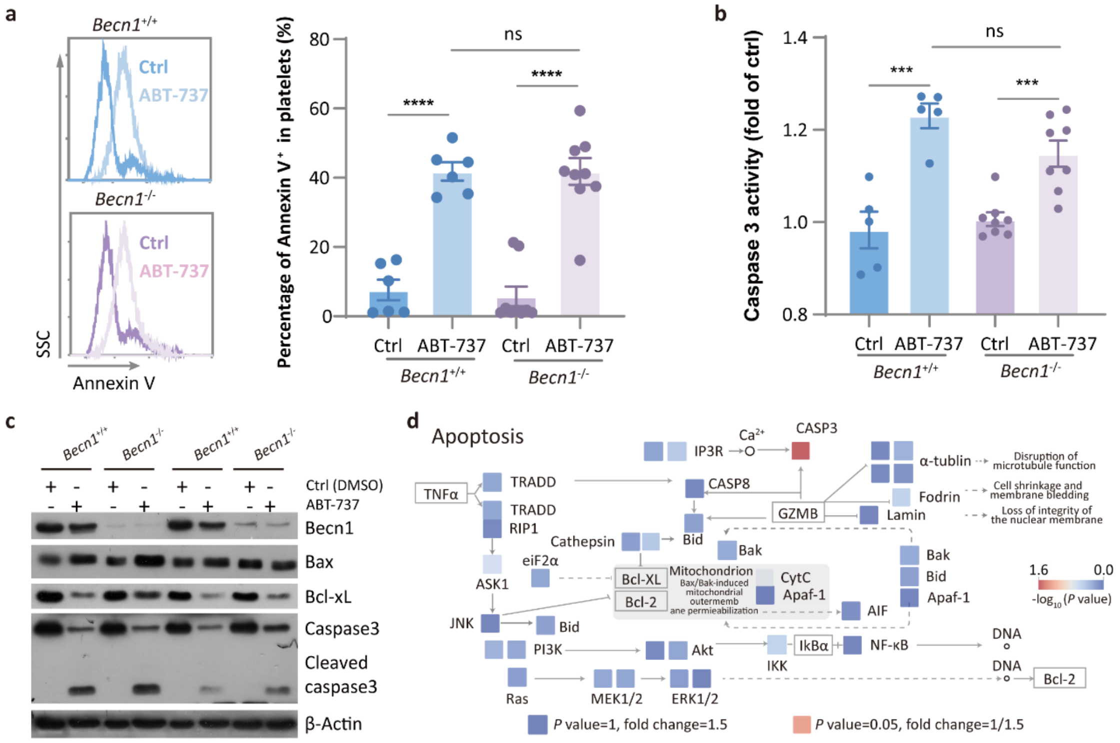

Figure S1. Pf4-Cre-mediated *Becn1* deletion does not increase apoptosis of platelets. (**a**) Flow cytometric analysis of platelet apoptosis after DMSO or ABT-737 treatment. (**b**) Spectrophotometric analysis of caspase 3 activity in platelets after DMSO or ABT-737 treatment. (**c**) Western blot analysis of proapoptotic or antiapoptotic proteins in platelets treated with DMSO or ABT-737. β-Actin was used as a loading control. (**d**) The proteins identified in the platelet proteome. The signaling pathway utilized in this study was adapted from the KEGG pathway mmu04210. The size of each node in the network was scaled to represent the magnitude of protein expression change in *Becn1*$^{f/f}$ and *Becn1*$^{f/f}$;Pf4-iCre mice. Furthermore, the color bar indicates the -log$_{10}$(P value), which reflects the statistical significance of the differences between the two groups. Data are mean ± SEM. ***P<0.001; ****P<0.0001.

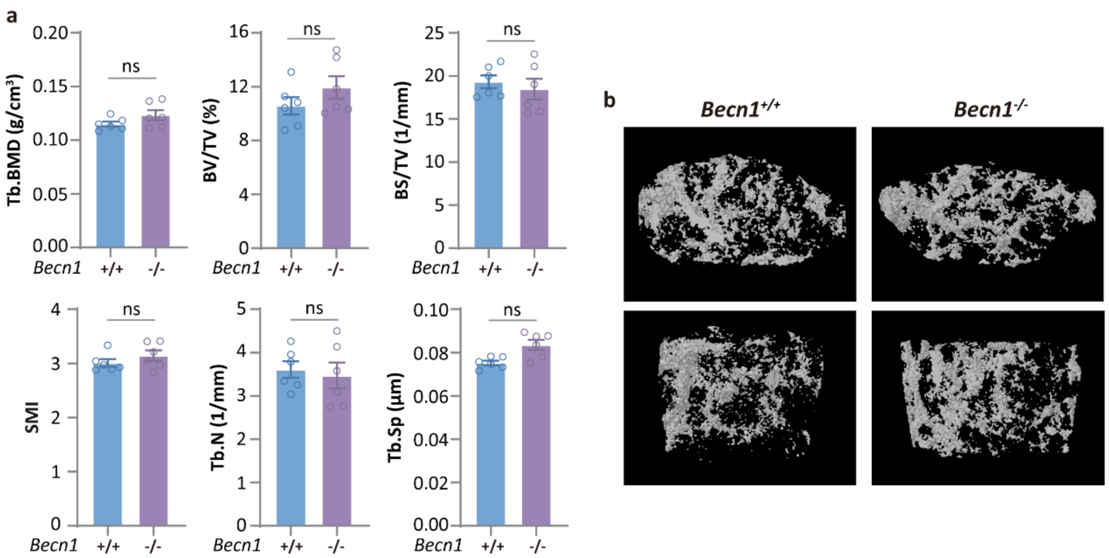

Figure S2. Micro-CT scanning of the femur trabecular and cortical bones of female *Becn1*$^{f/f}$;Pf4-iCre mice did not reveal any significant increase in bone mass. **(a)** Micro-CT analysis of female *Becn1*$^{f/f}$ and *Becn1*$^{f/f}$;Pf4-iCre mice. **(b)** Three-dimensional reconstructions of female *Becn1*$^{f/f}$ and *Becn1*$^{f/f}$;Pf4-iCre mice. The data are the mean ± SEM.

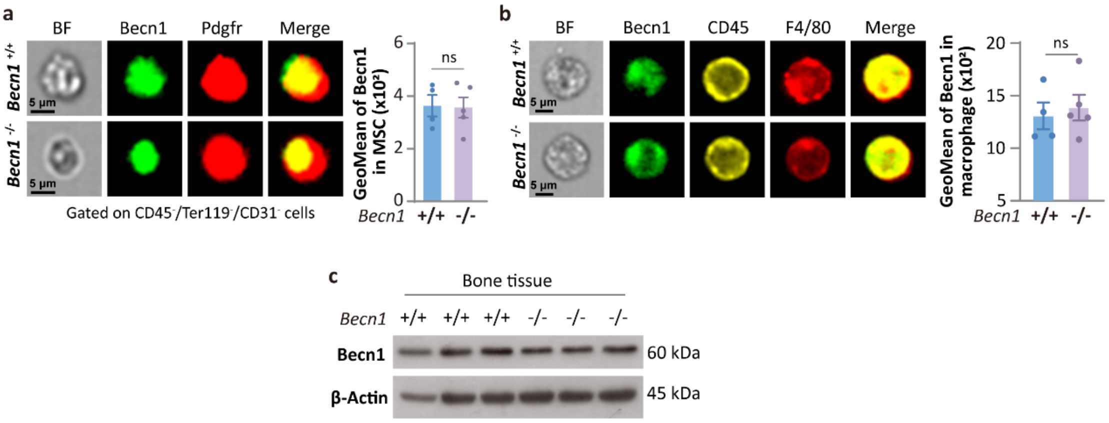

Figure S3. No significant changes in Becn1 were observed in mesenchymal stem cells, macrophages, and bone tissue of *Becn1*$^{f/f}$ and *Becn1*$^{f/f}$;Pf4-iCre mice. (a-b) Analysis of Becn1 expression (green) in bone tissue progenitor cells (MSCs, macrophages) was conducted using imaging flow cytometry in *Becn1*$^{f/f}$ and *Becn1*$^{f/f}$;Pf4-iCre mice. Left panel, representative flow image; right, results of flow image analysis. **(c)** Western blot analysis of Becn1 expression in bone tissue. The data are the mean ± SEM.

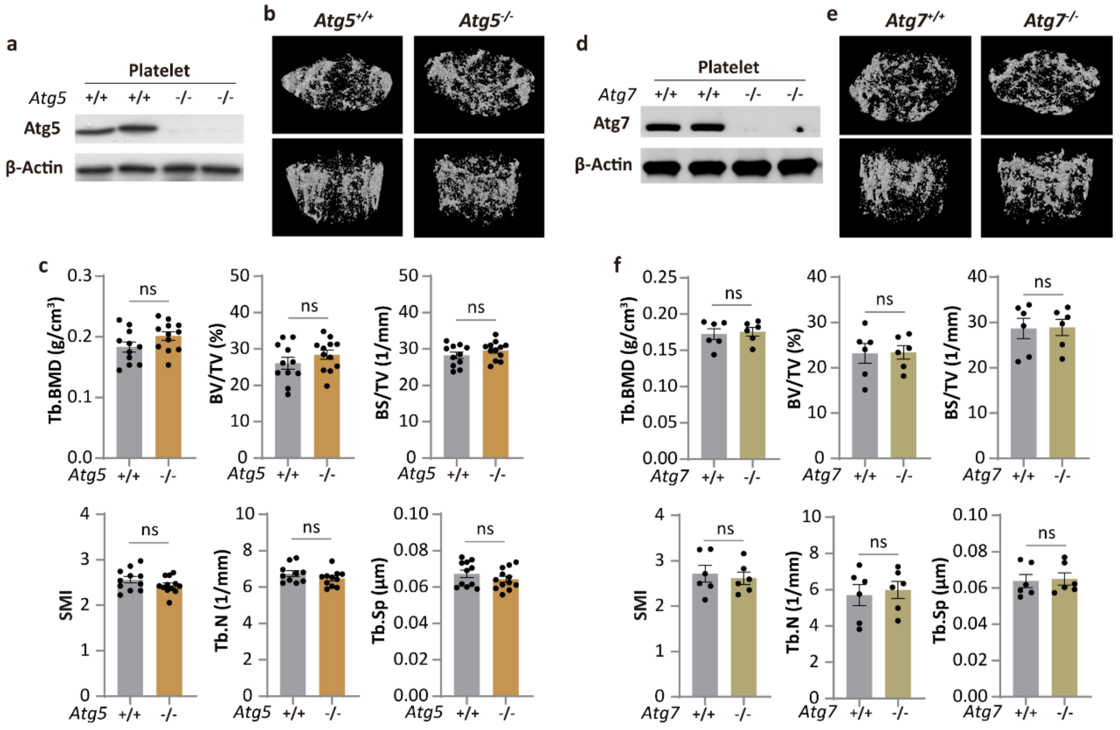

Figure S4. The loss of Atg5 or Atg7 in the megakaryocytic lineage does not affect bone mass. (a) Analysis of the Atg5 protein expression level in platelets by Western blotting. β-actin was used as a loading control. **(b-c)** Micro-CT analysis of the *Atg5*$^{f/f}$and *Atg5*$^{f/f}$;Pf4-iCre mice. **(d)** Analysis of the Atg7 protein expression level in platelets by western blotting. β-actin was used as a loading control. **(e-f)** Micro-CT analysis of the *Atg7*$^{f/f}$ and *Atg7*$^{f/f}$;Pf4-iCre mice. The data are the mean ± SEM.

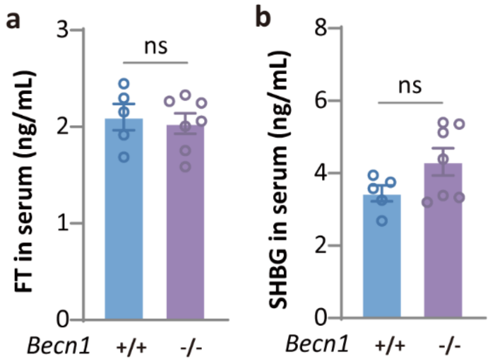

Figure S5. Deletion of *Becn1* in megakaryocytic lineage cells does not affect the levels of free testosterone and SHBG in the serum of female mice. (a) ELISA was performed to detect the levels of FT in the serum of female *Becn1*$^{f/f}$ and *Becn1*$^{f/f}$;Pf4-iCre mice. **(b)** ELISA was performed to detect the levels of SHBG in the serum of female *Becn1*$^{f/f}$ and *Becn1*$^{f/f}$;Pf4-iCre mice. The data are the mean ± SEM.

Table S1. Reagents used in this study

Reagent	Brand	Catalog
Thrombin	Chrono-log	P/N386
Prostaglandin E1	Enzo Life Science	GH0136
Hoechst	Invitrogen	H3570
Fibrinogen	Sigma	F3879
Alexa 594-conjugated phalloidin	Abcam	A12381
FITC anti-mouse CD2 Antibody	Biolegend	100105
FITC anti-mouse CD3 Antibody	Biolegend	100204
FITC anti-mouse CD5 Antibody	Biolegend	100606
FITC anti-mouse CD8a Antibody	Biolegend	100706
FITC anti-mouse TER-119/Erythroid Cells Antibody	Biolegend	116205
FITC anti-mouse Ly-6G/Ly-6C (Gr-1) Antibody	Biolegend	108406
FITC anti-mouse/human CD45R/B220 Antibody	Biolegend	103206
APC anti-mouse CD41 Antibody	Biolegend	133914
BD Horizon™ BV421 Rat anti-Mouse CD150	BD Biosciences	562811
Ly-6A/E (Sca-1) Monoclonal Antibody (D7), PerCP-Cyanine5.5	Invitrogen	45598182
CD117 (c-Kit) Monoclonal Antibody (2B8), PE	Invitrogen	12117182
CD45 Monoclonal Antibody (30-F11), PE-Cyanine7	Invitrogen	25045181
PE anti-Streptavidin Antibody	Biolegend	410504
FITC anti-mouse F4/80 Antibody	Biolegend	123108
Collagenase	Sigma	C2674
Dispase II	Sigma	D4693
Deoxyribonuclease I	Sigma	D5025
ABT-737	Selleck	S1002
BD Pharmingen™ FITC Annexin V	BD Biosciences	556419
Caspase-3 Assay Kit -Colorimetric	DOJINDO	C551

Beclin-1 (D40C5) Rabbit mAb	CST	3495S
Bax Antibody	CST	2772T
Bcl-xL (54H6) Rabbit mAb	CST	2764S
Caspase-3 Antibody	CST	9662S
Biotin N-hydroxysuccinimide ester	Sigma	h1759
Polyinosinic-Polycytidylic acid	Sigma	P0913
SHBG Antibody	HuaBio	ER1903-18
Atg5 Antibody	HuaBio	ET1611-38
Atg7 Antibody	HuaBio	ET1610-53
β-Actin Antibody	CST	4970T
